# Establishment of Methylation-Specific PCR for the Mouse p53 Gene

**DOI:** 10.4061/2011/938435

**Published:** 2011-12-12

**Authors:** Ryuji Okazaki, Akira Ootsuyama, Yasuhiro Yoshida, Toshiyuki Norimura

**Affiliations:** ^1^Department of Radiation Biology and Health, School of Medicine, University of Occupational and Environmental Health, 1-1 Iseigaoka Yahatanishi-ku, Kitakyushu 807-8555, Japan; ^2^Department of Immunology and Parasitology, School of Medicine, University of Occupational and Environmental Health, 1-1 Iseigaoka Yahatanishi-ku, Kitakyushu 807-8555, Japan

## Abstract

Methylation-specific PCR (MSP) of the mouse p53 gene has not yet been reported. We searched the CpG islands, sequenced the bisulfited DNA, and designed PCR primers for methylation and unmethylation sites. DNA from a young mouse produced a strong PCR product with the unmethylated primer and a weaker band with the methylated primer. DNA from an old mouse produced bands of similar intensities with both primers. In radiation-induced tumors, DNA from an old mouse yielded similar bands with both types of primers. We suggest that MSP is a valuable technique for the epigenetic study of the mouse p53 gene.

## 1. Introduction

A number of specific loci have been described as becoming hypermethylated during aging or carcinogenesis [1]. Most remaining CpG sites are normally methylated in adult cells. Transcriptional inactivation by cytokine methylation at promoter CpG islands of tumor suppressor genes is thought to be an important mechanism in human carcinogenesis [2]. In half of all primary human cancer cells, p53 is the most commonly mutated tumor suppressor gene [3]. Moreover, p73, a p53 homologue, has been shown to be hypermethylated in leukemia [4]. Using human cancer cells, Herman and coworkers [5] first described methylation-specific PCR (MSP), which can rapidly assess the methylation status of virtually any group of CpG sites. The MSP primer sequences for the human p53 gene have been identified in medulloblastomas [6], human gliomas [7], and hepatocellular carcinoma [8]. While there are many mouse experiments, the MSP primer sequences for the mouse p53 gene have yet to be reported. It is thought that it helps a future study very much to establish MSP technique. In this study, to establish MSP for this gene, we analyzed a CpG site in the mouse p53 gene and designed the corresponding primer sequences.

## 2. Materials and Methods

### 2.1. Experimental Animals

Mice carrying a disrupted, nonfunctional p53 gene (*p53^−/−^*) were derived from homologous recombination in an embryonic stem cell line from 129/SvJ mice as described previously [9, 10]. Wild type mice of the parental inbred strain were used as controls for the* p53^+/+^* mice. *p53^+/^* ^−^ mice were obtained by crossing male *p53^−/−^* mice with female *p53^+/+^* mice. The experimental protocols were approved by the Ethics Review Committee for Animal Experimentation of the University of Occupational and Environmental Health, Japan (Kitakyushu, Japan).

### 2.2. Irradiation

 A ^90^Sr-^90^Y disk delivered a beta ray source of 1.85 GBq at 15 Gy/min. The backs of seven-week-old mice (*p53^+/+^* and * p53^+/−^*) were irradiated with beta rays three times a week until the appearance of a tumor. These mice received 5 Gy/day.

### 2.3. Isolation of Genomic DNA

Genomic DNA from the spleen, liver, kidney, and tumor was isolated using a QuickGene DNA (Fujifilm Holdings Corporation, Tokyo, Japan) whole blood kit according to the manufacturer's instructions.

### 2.4. Bisulfite Conversion

Approximately, 1 *μ*g of DNA was treated with sodium bisulfite using an EpiTect Bisulfite kit (Qiagen, Hilden, Germany) according to the manufacturer's instructions. Briefly, the DNA, bisulfite mix, and DNA Protect buffer were mixed together. The bisulfite conversion thermal cycling conditions were as follows: 99°C for 5 min, 60°C for 25 min, 99°C for 5 min, 60°C for 85 min, 99°C for 5 min, and 60°C for 175 min. Finally, the bisulfite-converted DNA was purified on a spin column and eluted with 20 *μ*L of EB buffer.

### 2.5. Amplification of PCR Product

PCR primers were designed using Methyl Primer Express Software v1.0 (Applied Biosystems Inc. Foster City, CA, USA). PCR primer sets included sense (5′-ATC GTT ATT CGG TTT GTT TTC-3′) and antisense (5′-CAC GAC CTC CGT CAT ATA CT-3′) primers. Thermal cycling conditions were as follows: 1 cycle at 95°C for 10 min; 30 cycles at 94°C for 15 s, 55°C for 30 s, and 72°C for 30 s; 1 cycle at 72°C for 10 min.

### 2.6. Ligation, Transformation, and Sequence

PCR products were inserted into a pcDNA 3.1/V5-His-TOPO vector by TOPO cloning technology. The reaction was mixed gently and incubated for 5 minutes at room temperature. Two microliters of this reaction mixture were then added to a tube of TOP10 competent *E. coli* and mixed gently. After incubating on ice for 30 minutes, the cells were heat shocked for 30 seconds at 42°C without shaking. Tubes were immediately transferred back to the ice, and 250 *μ*L of room temperature medium was added. After incubation at 37°C for 30 minutes, reaction mixtures were spread onto a prewarmed ampicillin plate and incubated overnight at 37°C. Colonies were picked and cultured overnight in LB medium containing 50 *μ*g/mL ampicillin. We isolated the plasmid DNA using a miniprep kit (Qiagen), and its sequence was analyzed using both the T7 primer and reverse sequencing primers.

### 2.7. Sequence Analysis

DNA sequencing was performed with a BigDye terminator v3.1 cycle sequencing kit (Applied Biosystems Inc.) according to the manufacturer's instructions using the T7 sequencing primer (Invitrogen, Carlsbad, CA, USA) and the sense primer 5′-TAA TAC GAC TCA CTA TAG GG-3′. Thermal cycling conditions included 1 cycle at 96°C for 1 min and 25 cycles at 96°C for 10 s, 50°C for 5 s, and 60°C for 4 min. The DNA sequencing reactions were purified using the BigDye XTerminator purification kit (Applied Biosystems Inc.). The DNA sequencing reactions in 96 well plates were run on a 3130/3130xl genetic analyzer (Applied Biosystems Inc.), and resulting sequences were analyzed with sequencing analysis software (Applied Biosystems Inc.).

### 2.8. Methylation Specific PCR (MSP)

We searched for CpG islands in the p53 gene and designed PCR primer sets using Methyl Primer Express Software v1.0 (Applied Biosystems Inc.). PCR amplification was performed using an EpiTect MSP kit (Qiagen). Thermal cycling conditions were as follows: 1 cycle at 95°C for 10 min; 35 cycles at 94°C for 15 s, 50°C for 1 min and 72°C for 30 s; 1 cycle at 72°C for 10 min.

## 3. Results and Discussion

### 3.1. Mapping of DNA Methylation Patterns in a CpG Islands of the Mouse p53 Gene and the Sequence of Bisulfited DNA

**Figure 1:**
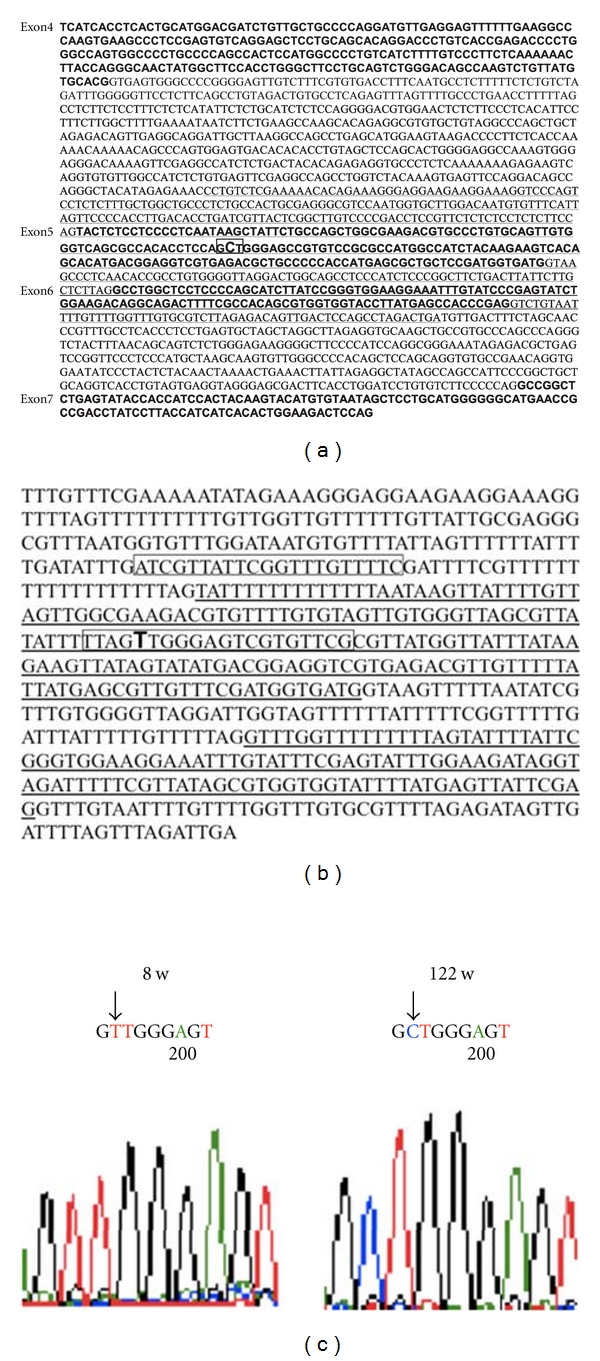
(a) CpG islands of the mouse p53 gene from exon 4 to exon 9. Underlined sequences indicate CpG islands that were searched using Methyl Primer Express Software v1.0. The cytosine (C) surrounded by a rectangle is the CpG site. (b) The sequence of the CpG islands after bisulfite conversion. The boxed area indicates the forward and reverse primer sets. Underline sequences refer to exons 5 and 6. (c) Electropherograms of PCR products after subcloned plasmid DNA in 8-week-old and 122-week-old *p53^+/+^* mice. In 8-week-old mice, a “C” was converted to a “T”. In 122-week-old mice, a “C” was retained as a “C”.

We searched the mouse p53 gene from exon 4 to 9 for a CpG islands using Methyl Primer Express Software v1.0 (Figure 1(a); Applied Biosystems Inc.) and found one located from intron 5 to intron 7, spanning 619 base pairs. The cytosine (C) surrounded with a rectangle is the specific CpG site (Figure 1(a)). This is the first description of a CpG island for the p53 gene in normal mouse tissues. Using the same software, the CpG island was searched with simulated bisulfite-converted DNA sequences. The resulting sequences were the same as the sequences that were analyzed by the genetic analyzer in this study.

Several candidates for PCR primer sets were given by the software, and the suitable set, determined after many rounds of PCR, included a methylated base. The best sets for p53-unmethylation and p53-methylation are surrounded by a rectangle in Figure 1(b) and given in Table 1. Cytosine (C) of GCT (Alanine) in exon 5 was converted to a thymidine (T) after bisulfite treatment in young mice, which did not occur in old mice after the same treatment (Figures 1(b), 1(c)).

### 3.2. MSP Analysis

**Figure 2:**
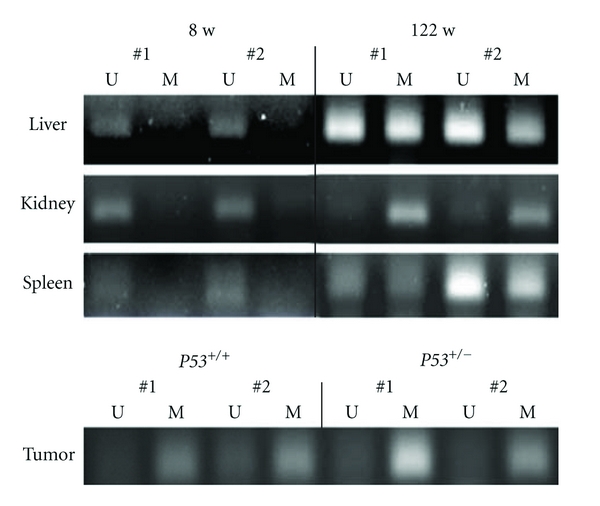
MSP analysis. Primer sets used for amplification are designated as unmethylated (U) and methylated (M). Liver, kidney, and spleen samples were from two 8-week-old mice and two 122-week-old *p53^+/+^* mice. Tumors were induced by a ^90^Sr-^90^Y beta ray in* p53^+/+^* and *p53^+/−^* mice.

In all organs (i.e., liver, kidney, and spleen) of 8-week-old mice, we obtained a strong PCR product using the unmethylated primer and weaker band with the methylated primer. Alternatively, in liver and spleen from 122-week-old mice, the DNA was amplified to similar PCR products with both the unmethylated and methylated primers (Figure 2).

In radiation-induced tumors from *p53^+/+^* mice and *p53^+/−^* mice, amplified DNA yielded similar bands with both the methylated and unmethylated primers (Figure 2) except in one tumor from a *p53^+/−^* mouse, which showed a strong band with the former and a weaker band with the latter.

We have previously shown data for MSP in the *p53* gene [11] where *p53* methylation increased with age in the spleen after irradiation at a young age. After DNA damage is induced by intra- and/or extracellular processes, DNA methylation is known to increase with increasing age [12]. In this study, we have shown how *p53* methylation in the spleen and liver relates to age and that *p53* methylation was found in all radiation-induced tumors in mice. Finally, it is interesting to note that methylation increases in tumor suppressor genes in human tumors as well [13], including the *p53* gene [6–8].

In this study, we established MSP for the mouse p53 gene, and this is the first description. Unfortunately, we were only able to find one base (C) that was methylated, observing bands of similar intensity using both unmethylated and methylated primers. Therefore, there is still room for improvement in this MSP method. However, we could show MSP technique for mouse p53. The technique is so simple and easy. It is thought that the width of the methylation study will progress in future by MSP technique for the mouse p53 gene having been established.

## Figures and Tables

**Table 1:** Primer sequences for p53 MSP analysis.

		Primer sequence (5′–3′)
U^1^	F^3^	ATC GTT ATT CGG TTT GTT TTC
	R^4^	CGA ACA CGA CTC CCA ACT AA
M^2^	F	ATC GTT ATT CGG TTT GTT TTC
	R	CGA ACA CGA CTC CCA GCT AA

^1^U: un-methylated sequence; ^2 ^M: methylated sequence; ^3^F: forward sequence; ^4^R: reverse sequence.
